# Safe Removal of the Urethral Catheter 2 Days Following Laparoscopic Radical Prostatectomy

**DOI:** 10.5402/2012/912642

**Published:** 2012-08-17

**Authors:** Philip James, Anthony Glackin, Alan Doherty

**Affiliations:** Queen Elizabeth Hospital, University Hospitals Birmingham NHS Foundation Trust, Edgbaston, Brimingham B15 2TH, UK

## Abstract

*Purpose*. To assess the risks and benefits of early urethral catheter removal following laparoscopic radical prostatectomy. *Materials and Methods*. Between June 2009 and April 2011, 114 patients underwent laparoscopic radical prostatectomy for clinically organ-confined prostate cancer. Candidates for early removal of the urethral catheter were selected intraoperatively on the basis of the integrity of the vesicourethral anastamosis and the ease of recatheterisation. In the selected cohort of patients, the urethral catheter was removed at day 2. Recatheterisation rates within this group were recorded and analysed. *Results*. Of the 114 patients who underwent laparoscopic prostatectomy, 64 (56%) were deemed suitable for removal of catheter on second postoperative day prior to discharge. The first 20 patients selected for early removal of urethral catheter were covered with a suprapubic catheter inserted at the time of surgery. Out of 64 patients deemed suitable for early removal of urethral catheter, 53 (83%) were able to pass urine without complication. 11 patients (17%) developed urinary retention that necessitated recatheterisation. In all cases, reinsertion of catheter was performed easily and successfully without the need for cystoscopic guidance or adjuncts. *Conclusions*. Removal of the urethral catheter at day 2 following laparoscopic prostatectomy is a safe procedure in carefully selected patients.

## 1. Introduction

Laparoscopic radical prostatectomy has gained worldwide acceptance as a treatment for organ-confined prostate cancer since the first feasibility report by Schuessler et al. [1] in 1997, and the standardisation of the technique by Guillonneau et al. [2] in 1999. Advantages of this minimally invasive approach have been cited by multiple studies and include short hospital stay, better pain control, and faster return to everyday activities [3].

One of the proposed benefits of laparoscopic prostatectomy also includes reduced catheterisation time [4]; however, the duration of catheterisation varies greatly between surgeons and institutions. This retrospective series describes our experience of removing the urethral catheter only 2 days following laparoscopic radical prostatectomy.

This is the first assessment in the literature of the safety of catheter removal two days after laparoscopic prostatectomy, without the need for cystography [5]. Most surgeons who remove the catheter early continue to obtain a cystogram to ensure healing [6]. We intend to show that early removal of catheter is a safe procedure, and that careful intraoperative selection of candidates obviates the need for a cystogram prior to catheter removal.

## 2. Patients and Methods

114 patients underwent laparoscopic radical prostatectomy, by an experienced surgeon, for clinically organ-confined prostate cancer at our institution between June 2009 and April 2011. Prior to surgery, no patient had an indwelling urinary catheter. Laparoscopic prostatectomy was performed under general anaesthetic using an extraperitoneal 5-port approach. The posterior aspect of the rhabdosphincter was restored with the technique described by Rocco et al. [7]. The vesicourethral anastamosis and any required bladder neck reconstruction were performed with continuous 2-0 Vicryl (Ethicon) suture tied intracorporeally after exposure of the urethral stump by a Foley catheter. In all patients, bilateral nerve sparing was performed whenever possible according to preoperative factors, such as clinical stage of the cancer, and preoperative potency as determined by use of the Sexual Health Inventory for Men (SHIM) score.

Patients deemed suitable for trial without catheter two days after laparoscopic prostatectomy were selected intraoperatively. Inclusion criteria for early catheter removal included water-tight anastamosis, and easy reinsertion of urethral catheter at the time of surgery. Complicating factors such as previous radiotherapy or any previous prostatic, bladder neck, urethral or pelvic surgery, were not viewed as definite exclusion criteria.

Within the cohort suitable for early removal of urethral catheter, the initial 20 cases were covered with a suprapubic catheter inserted intraoperatively. Given that we noted no untoward sequelae regarding recatheterisation when necessary and as our experience with the technique matured we felt secure in abandoning the need for a suprapubic catheter in the remaining patients.

Once the cohort had been identified, the results of the trial without catheter were recorded. If the trial without catheter failed, the urethral catheter was reinserted by a urologist. The patients who failed to void following removal of their catheter were recatheterised and discharged home to return on postoperative day 7 for a second trial without catheter as an out patient.

## 3. Results

Of the 114 patients who underwent laparoscopic prostatectomy, a total of 64 patients (56%) were deemed suitable at the time of surgery for early removal of the urethral catheter. This was performed on day 2 postoperatively. The first 20 patients within this cohort (31%) also received a suprapubic catheter intra-operatively to allow an alternative route for the drainage of urine if it should be required.

Of the 64 patients who underwent early removal of urethral catheter on day 2, 53 patients (83%) passed urine perurethra without complication. 11 patients (17%) suffered urinary retention that necessitated recatheterisation. This was performed on the ward by a urology resident without complication or need for cystoscopic guidance/adjuncts.

## 4. Discussion

The development of laparoscopic techniques has revolutionised the management of malignant disease across almost all surgical specialities. The first case series looking at the feasibility of laparoscopic prostatectomy reported a mean operating time and length of hospitalisation of 9.4 hours and 7.3 days, respectively [1]. The evolution of minimally invasive techniques for the treatment of prostate cancer has seen a substantial decrease in both operative time and convalescence, in addition to reports of improvements in postoperative pain [8] and duration of catheterisation [5]. These improvements have been attributed to the development of intracorporeal suturing techniques and improved instrumentation, in addition to the improved visualisation of the vesicourethral anastamosis that laparoscopy provides. It is these advances in technique that have allowed us to challenge previous postoperative management plans.

From the perspective of a patient's quality-of-life, early catheter removal provides an advantage following laparoscopic prostatectomy. Bladder catheterisation produces symptoms similar to those of an overactive bladder, with involuntary muscarinic-receptor-mediated contractions [9]. This discomfort exacerbates postoperative pain, and is resistant to conventional therapy [10]. In addition to an individual's discomfort, prolonged catheterisation has been associated with the history and pathology of urethral stricture disease [11]. Although early catheter removal is unlikely to produce a significant decrease in stricture formation rates following prostatectomy, most patients would prefer to be free of the catheter prior to discharge. In addition, a trial without catheter as an outpatient needs considerable nursing input and is a burden on health care resources [12].

This study has shown that it is safe to remove the urethral catheter at day 2 following laparoscopic prostatectomy in carefully selected patients. These patients must have a watertight anastamosis which must be able to admit a catheter without the use of an introducer or flexible cystoscope. These conditions can be easily and accurately assessed at the end of the operation. If recatheterisation was required, it was always performed on the ward with no difficulty, and without the need of any adjuncts. No cases needed urological input. Concerns regarding early catheter removal and the presumed risk to the vesicourethral anastamosis associated with recatheterisation (e.g. creating false passages or undermining the bladder neck) were not realised in our cohort of patients. These results are equivalent to several contemporary open prostatectomy series which have also reported success with early catheter removal [13]. The risk of early catheter removal in carefully selected cases is minimal, and as such we believe that the benefits to the patient make this a suitable and desirable practice.
